# Excessive distal migration of fiber-mesh coated femoral stems: A randomized RSA study of 41 hips

**DOI:** 10.3109/17453674.2011.574562

**Published:** 2011-07-08

**Authors:** Thomas Baad-Hansen, Søren Kold, Niels Olsen, Finn Christensen, Kjeld Søballe

**Affiliations:** ^1^Department of Orthopaedic Surgery, Aarhus University Hospital, Aarhus; ^2^Department of Orthopaedic Surgery, Kolding Region Hospital, Kolding, Denmark

## Abstract

**Background:**

The surface texture, localization, and magnitude of the surface material applied to the femoral stem can facilitate bone ingrowth and influence the survival of total hip arthroplasties. Clinical and radiographic studies have shown superior bone ingrowth in proximally porous-coated stems with a diaphyseal grit-blasted surface in comparison to a smooth diaphyseal surface. Surface textures—especially porous surface material—have been suggested to have a sealing effect against migration of polyethylene debris along the implant-bone interface and to reduce the inflammatory response, leading to a prolonged implant survival.

**Patients and methods:**

Between 2004 and 2006, we conducted a randomized, controlled trial (RCT) involving 50 patients with non-inflammatory arthritis. They received either a distally tapered, extended coated stem or a straight, proximally coated stem. During surgery, tantalum markers were inserted into the greater and lesser trochanter. Implant migration was evaluated at 3, 12, and 24 months postoperatively by radiostereometric analysis. The primary endpoint was stem migration 2 years after surgery.

**Results:**

All femoral components in both groups showed pronounced distal translation, with the highest rate of translation occurring between 0 and 3 months. After 2 years, the mean distal translation was 2.67 (95% CI: –3.93 to –1.42) mm for the tapered, extended coated stem and 1.80 (–2.45 to –1.15) mm for the straight, proximally coated stem. Half of the tapered, extended coated stems and two-thirds of the straight, proximally coated stems had migrated more than 1 mm. No difference between the 2 stems could be seen with regard to translation or rotation at any time point. After 2 years, 2 hips have been reoperated due to mechanical loosening of the stem.

**Interpretation:**

An excessive amount of migration of both stem types was seen 2 years postoperatively. It is of vital importance to follow this patient cohort since radiostereometric analysis is known to be predictive of late implant failure, especially in this study where pronounced early migration was observed. We recommend longer follow-up of both stem types.

Uncemented femoral stems were introduced more than 3 decades ago (Hansen and Rechnagel 1977, Brown and Ring 1985) and today they are the most frequently used femoral implants in total hip arthroplasty (THA) in Denmark (Lucht and Johnsen Paaske 2004). Achievement of immediate postoperative bone-implant contact is essential for the initial stability of the implant (Soballe et al. 1992) and distal stem geometry influences the initial stability. In one experimental study, a fluted, tapered stem design showed higher tolerable initial axial and torsional loads than a cylindrical design (Kirk et al. 2007). Also, the surface texture, localization, and magnitude of surface material can facilitate bone ingrowth and influence the survival of uncemented THAs. Experimental studies have shown that blasted titanium surfaces support extensive bone growth directly onto the implant (Feighan et al. 1995, Rahbek et al. 2005) and ingrowth can be enhanced by addition of a porous coating (Overgaard et al. 1998). Furthermore, stems with a proximally porous-coated surface with an additional diaphyseal grit-blasted surface have been reported to have better fixation and a lower degree of osteolysis than a smooth diaphyseal surface (Kang et al. 2000). Surface textures—especially porous surface material—have been suggested to have a sealing effect against migration of polyethylene debris along the implant-bone interface, reducing the inflammatory response (Bobyn et al. 1995) and reducing the risk of implant failure. 2 recent studies of a tapered, extended coated stem identical with the stem tested in the present study found no stem subsidence greater than 2 mm and no revision at a mean follow-up of 7 years (Klein et al. 2009). At 5-year follow-up, Akhavan and Goldberg (2007) observed bony integration radiographically in all 100 stems implanted.

In this study, our hypothesis was that stems with extended coating would achieve a better degree of fixation over a larger area, leading to better initial stability. Stem subsidence exceeding an upper limit of 1–2 mm 2 years after hip surgery has been found to be correlated with an increased risk of implant failure in later years (Karrholm et al. 1994, Kobayashi et al. 1997). We therefore designed an RCT to compare the migration and rotation of a distally tapered, extended coated stem with that of a straight, proximally coated stem using radiostereometric analysis (RSA). The primary endpoint was stem migration 2 years after surgery.

## Patients and methods

### Recruitment, randomization, and sample size

Between 2004 and 2006, we randomized 50 patients to receive one of 2 types of femoral stems: a distally tapered, extended coated stem or a straight, proximally coated stem. Patients were included in the study if (1) they had primary osteoarthritis, (2) they had sufficient bone density to allow uncemented implantation of a femoral component, and (3) they had no regular intake of non-steroidal anti-inflammatory drugs. 50 sealed, opaque envelopes contained one or other choice of implant to be inserted. After commencing surgery, an envelope was drawn by a scrub nurse and this decided which of the 2 implants should be used. There were 25 men and 25 women. Median age was 60 (49–70) years.

**Figure 1.**
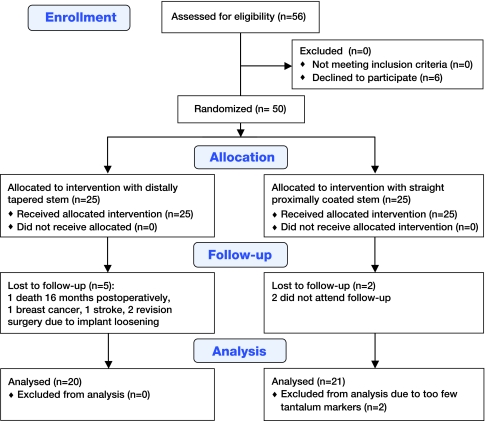
Flow chart showing the recruitment and randomization strategy.

Based on an estimated clinically important difference in stem migration of 0.6 mm and an SD of 0.7 mm between groups (Ryd 1992), the pre-study sample size calculation indicated that 22 patients would be required in each group to achieve a power of 80% at the 5% significance level. Due to the possibility of patient dropout, 25 patients were included in each group. One patient died 16 months after surgery, and 2 patients suffered from diseases unrelated to the THA (breast cancer and stroke) and could not continue radiographic follow-up. 2 patients did not wish to attend radiographic follow-up. 2 patients were excluded due to insufficient numbers of tantalum markers inserted in the adjacent bone, making radiostereometric analysis impossible. Finally, 2 patients were reoperated during the follow-up period. This left 41 patients (41 hips) for analysis. The recruitment and randomization strategy is shown in Figure 1. The trial was registered at ClinicalTrials.gov (reg. no. NCT00116051). The design and procedures of the clinical trial were approved by the local ethics committee before any patients were included, and it was performed in accordance with the Helsinki Declaration (II). Written informed consent was obtained from all patients. Additional permission to perform 10 double RSA examinations was granted by the local ethics committee.

### Implant design

**Figure 2.**
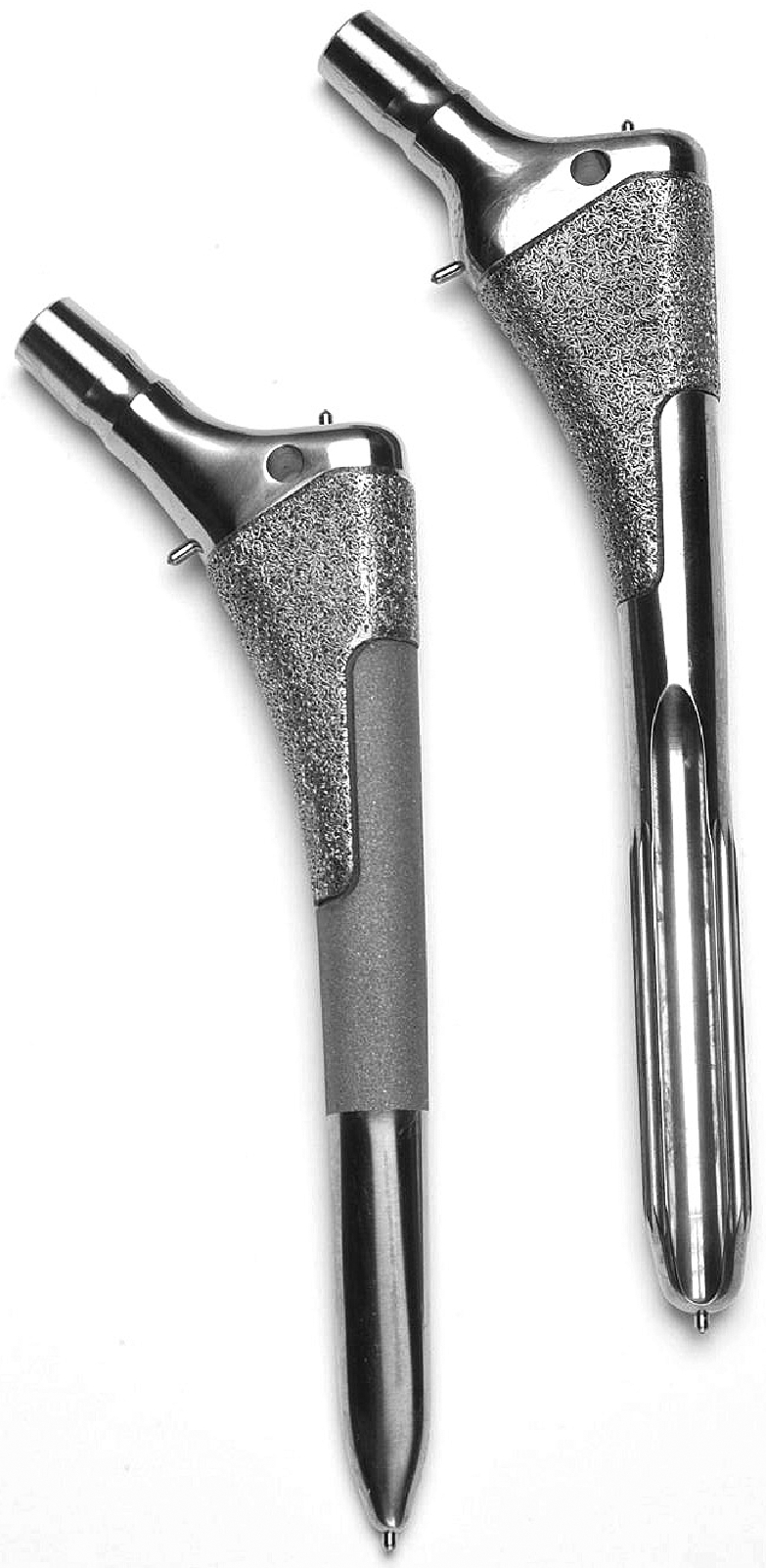
The distally tapered, extensively coated stem (VerSys Fiber Metal Taper) (left) and the straight, proximally coated stem (VerSys Fiber Metal MidCoat) (right).

Patients were randomized to receive a distally tapered, extended coated stem (VerSys Fiber Metal Taper Hip Prosthesis (FMT); Zimmer Inc., Warsaw, IN) or a straight, proximally coated stem (VerSys Fiber Metal MidCoat Hip Prosthesis (FMM); also Zimmer) (Figure 2). Both of these femoral components were uncemented implants. The FMT has a circumferential titanium fiber-mesh surface located proximally, a midshaft circumferential corundum surface extending longer laterally, and a polished, tapered distal part. The FMM also has a circumferential titanium fiber-mesh surface located proximally, identical to the FMT, but has a polished distal part with splines and flutes. The manufacturer fastened 1.0-mm tantalum markers at the distal tip, at the medial side, and at the lateral top of both types of femoral components. All patients received a 28-mm cobalt-chromium alloy head. The patients received a Mallory-Head acetabular component (Biomet Inc., Warsaw, IN) with the exception of 5 patients who received an uncemented Saturne cup (Amplitude, Neyron, France); 2 and 3 in the 2 groups.

### Surgical technique

2 senior surgeons (NO and FK) performed all the operations. Preoperatively, an analog templating was performed using a transparent drawing of the prosthesis with a 20% magnification fitted on antero-posterior radiographs of both hips. A posterolateral approach (the Moore approach) was used. Femoral reaming was performed according to the manufacturer's guidelines, starting with intramedullary reamers 4 mm smaller than the anticipated prosthesis size. The reaming procedure was continued until the desired canal diameter was obtained. Femoral rasps were introduced with increasing size, beginning with rasps 2 sizes smaller than the expected femoral stem size. The rasping procedure was continued until the rasp filled the femur adequately, based on preoperative templating and intraoperative evaluation, in an attempt to avoid perioperative fractures of the femur or over-reaming of the canal. The implant was pressed down the canal by hand until no further advancement was possible, corresponding to where the proximal portion of the porous coating was even with the neck osteotomy. Correct stem rotation was ensured; a stem impactor was inserted into the implant slot and tapped with a mallet until the stem was fully seated. Initial stability of the implant was tested manually during the operation; no measurement of axial and torsional load was done.

Intraoperatively, four 1.0-mm tantalum markers were inserted in the major trochanter and 4 markers were inserted in the minor trochanter. These markers formed a reference rigid body.

Full weight bearing was allowed immediately after the surgery, using 2 crutches for the first 2 weeks and a single crutch over the next 6 weeks. During admission, all patients received supervised specialist training by the departmental physiotherapists.

### Evaluation

All patients were clinically evaluated using the Harris hip score at 3 months and at 12 months postoperatively. Antero-posterior and lateral radiographs were taken postoperatively, and also at 3 months and 12 months after the operation.

**Figure 3.**
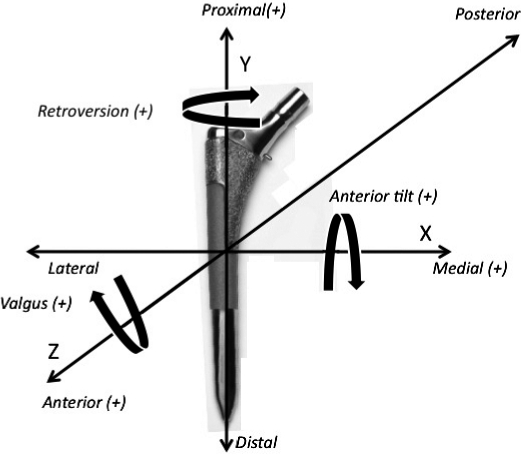
The coordinate axes of stem rotation and translation.

RSA examinations were performed within the first week after surgery and after initial mobilization, and again at 3 months, 12 months, and 24 months after surgery. A calibration box (Large Calibration Box Aarhus; Medis, Leiden, the Netherlands) was placed beneath the patient, holding 2 phosphorus plates using the uniplanar technique (Selvik 1990). 2 stationary radiographic tubes were placed in such a position that the X-ray beams crossed at an angle of approximately 40°. The exposure was set to 150 kV and 3 mAs. We used Marker-Based RSA version 3.2 (Medis Specials, Leiden, the Netherlands) to calculate the implant migration. Total stem migration was calculated as √(x^2^ + y^2^ + z^2^), where x, y, and z are the Cartesian components of translation. The coordinate axes of the stem rotation and translation are shown in Figure 3.

10 patients underwent 2 examinations to assess the precision of the RSA system. Between the 2 investigations, the equipment was removed from the location and repositioned within 5–10 min. Test-retest examinations were performed at the 24-month follow up. The precision of the translation and rotation was calculated from the double examinations and was below 0.2 mm (anterior translation) and 0.98° (longitudinal rotation) (Table 1). The average condition number (defined as the geometric distribution of tantalum markers in 3 dimensions) was calculated to be 22, indicating that the tantalum markers inserted in the surrounding femoral bone were distributed sufficiently to be able to obtain appropriate data for determination of prosthetic migration (Valstar et al. 2005).

**Table 1.** Double examination of 10 patients. The precision is presented as the mean absolute value + 2 SD (95% CI for significant movement)

	Stem migration
Translation	
Medial-lateral (x)	0.12 mm
Proximal-distal (y)	0.10 mm
Anterior-posterior (z)	0.19 mm
Rotation	
Anterior(+) / posterior (-) tilt	0.41°
Ante(-) / retroversion (+)	0.98°
Valgus (+) / varus (-)	0.62°

### Statistics

We performed probability plots (Q-Q plots) to test for normality of the migration and rotation data. We determined differences in migration and rotation between the distally tapered, extended coated stem and the straight, proximally coated stem using repeated-measurements analysis of variance (ANOVA) to compare RSA measurements postoperatively, and at 3, 12, and 24 months. To compare migration and rotation between groups at a given time, t-test was used. Statistical analysis was performed with the STATA 8 software package.

## Results

**Figure 4.**
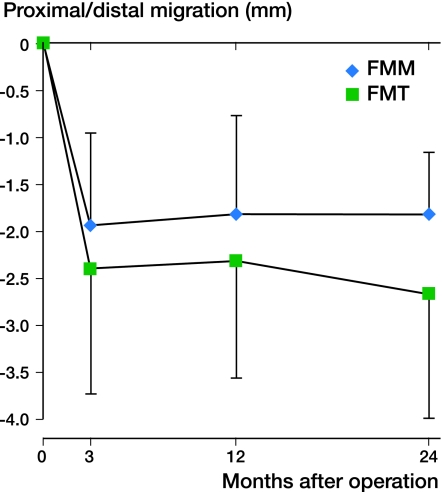
Mean distal migration (in mm) of the FMM and FMT femoral components. Migration values are mean with 95% confidence interval.

**Figure 5.**
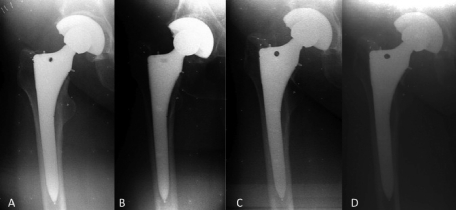
Distal migration of an FMT stem. A. Immediately postoperatively. B. At 3 months. C. At 12 months. D. At 24 months.

All femoral components in both groups had a pronounced translation in the distal direction within the femur. 7 of the 41 stems migrated so far distally that the marker attached to the medial side of the stem was disconnected from the prosthesis because of direct contact with the calcar, leading to inconclusive stem rotation results. The highest rate of translation occurred between 0 and 3 months (Table 2). At the 2-year follow-up, the mean distal translation was 2.67 (95% CI: –3.93 to –1.42) mm for the FMT and 1.80 (–3.93 to –1.42) mm for the FMM, respectively (Figure 4). After 2 years, 10 of 20 stems in the FMT group and 14 of 21 stems in the FMM group had migrated more than 1 mm (Figure 5). The mean amount of total stem migration after 2-year follow-up was 3.28 (0.11–6.67) mm for the FMT stem and 2.73 (1.48–3.98) mm for the FMM stem. No statistically significant differences between the 2 stems could be seen with regard to translation (p = 0.2) or rotation (p = 0.2) at any time point.

**Table 2.** Stem translation and rotation at 3, 12, and 24 months. Absolute values are shown as mean with 95% confidence interval

	3 months (n=49)		12 months (n=46)		24 months (n = 41)	
	Mean	95% CI	Mean	95% CI	Mean	95% CI
Translation (mm)						
Medial(+) / lateral (-)						
FMT	0.13	-0.21 to 0.47	0.07	-0.31 to 0.44	0.12	-0.10 to 0.35
FMM	-0.04	-0.31 to 0.22	-0.26	-0.60 to 0.08	-0.08	-0.75 to 0.58
Proximal(+) / distal (-)						
FMT	-2.41	-3.66 to -1.18	-2.32	-2.98 to -0.64	-2.67	-3,93 to -1.42
FMM	-1.95	-2.93 to -0.31	-1.82	-2.88 to -0.77	-1.80	-2.45 to -1.15
Anterior(+)/ posterior (-)						
FMT	-0.51	-0.89 to -0.14	-0.13	-0.55 to 0.30	0.07	-0.52 to 0.67
FMM	-0.20	-0.49 to 0.10	-0.02	-0.43 to 0.39	-0.31	-1.15 to 0.52
Total migration						
FMT	2.75	1.42 to 4.09	2.78	-0.59 to 6.14	3.28	0.11 to 6.67
FMM	2.20	1.24 to 3.17	2.80	1.67 to 3.93	2.73	1.48 to 3.98
Rotation (°)						
Anterior(+) / posterior (-) tilt						
FMT	-0.35	-0.72 to 0.02	-0.54	-1.06 to -0.02	-0.94	-1.44 to -0.44
FMM	0.23	-0.60 to 1.05	-0.49	-1.41 to 0.44	-0.62	-1.90 to -1.67
Ante(-) / retroversion (+)						
FMT	-1.63	-3.89 to 0.62	0.34	-1.98 to 2.65	1.86	0.17 to 3.55
FMM	-0.56	-1.55 to 0.43	0.43	-1.45 to 2.30	1.29	-0.85 to 3.44
Valgus (+) / varus (-)						
FMT	0.16	0.01 to 0.32	-0.07	-0.26 to 0.11	-0.09	-0.28 to 0.10
FMM	-0.07	-0.44 to 0.29	0.43	-0.60 to 1.45	-0.19	-0.68 to 0.30

There was no statistically significant difference between surgeons regarding stem translation or rotation in any direction at the 2-year follow-up (p = 0.3).

2 patients were reoperated after 6 and 14 months because of implant loosening. The patient who underwent reoperation at 6 months had a distal migration of 10 mm at the RSA examination at 3 months. The other patient had a distal migration of 1.4 mm at 3 months and of 1.9 mm at 12 months by RSA.

## Discussion

The migration pattern of femoral components is complex, and may vary from stem type to stem type. Plausible reasons for this are stem design and surface texture. Early migration of femoral stems has been shown to foreshadow late failure (Freeman and Plante-Bordeneuve 1994, Karrholm et al. 1994, Kobayashi et al. 1997). Karrholm et al. (2002) evaluated 2 different uncemented stems with RSA; they found subsidence and rotation values close to zero at 2 years and no failures were observed during this time. On the other hand, 2 other studies (Karrholm et al. 1994, Derbyshire and Porter 2007) (Table 3) found that pronounced migration within the first 2 years after THA led to revision rates of between 10% and 50%.

**Table 3.** Migration and revision in different RSA studies

Stem type (references)	No. of hips	Observation time (years)	Distal migration (mm)	Revised hips during the observation time	Additional follow-up: Hips revised due to aseptic loosening
Cemented Elite Plus (Derbyshire and Porter 2007, Hauptfleisch et al. 2006)	25	3	0.3 (0.02–1.28) **^a^**	4%	9 years, 10%
Cemented C-stem (Sundberg et al. 2005)	33	2	1.35 (0.62) **^b^**	Not reported	
Cemented Exeter (Alfaro-Adrian et al. 2001)	32	1	0.86 (0.11) **^c^**	Not reported	
Cemented Lubinus SP I (Karrholm et al. 1994)	84	2	1.3–15.9 **^d^**	0%	7 years, 50% (subgroup revised)
Uncemented Epoch & Anatomic (Karrholm et al. 2002)	68	2	E: 0.05 (–0.16 to 1.04) **^a^** A: –0.02 (–0.31 to 0.31) **^a^**	0%	
Uncemented Ribbed stem (Karrholm and Snorrason 1993)	18	2	1.06 (0.4–2.1) **^a^**	6% (Indication: pain)	
Uncemented VerSys (Current study)	41	2	FMT: 2.67 (3.13) **^b^** FMM: 1.80 (1.52) **^b^**	4%	

**^a^** Mean and range

**^b^** Mean and SD

**^c^** Mean and SE

**^d^** Range

The Exeter stem (Table 3) has shown a distal translation of approximately 0.8 mm in the first year after surgery without signs of failure (Alfaro-Adrian et al. 2001). In contrast to the Exeter stem, the VerSys stem is not designed to subside, but to obtain initial stability. Karrholm et al. (1997) suggested that the amount of acceptable migration of uncemented femoral implants should be less than 1–1.5 mm within 2 years. However, in our study the mean distal translation was almost 2 mm after 3 months. A tendency of the stem migration to “level off” could be seen by comparing the migration after 3 months with that after 12 and 24 months. Distal translation unavoidably led to translation along the x- and z-axes and elevated stem rotation around all 3 axes because translation and rotation parameters are mutually dependent.

In experimental studies (Rahbek et al. 2005) and clinical studies (Thanner et al. 1999), the fiber metal porous coating used on the stems in our study exhibited high levels of osseointegration. Akhavan and Goldberg (2007) retrospectively reviewed 100 consecutive hips with the same fiber metal coated tapered stem and reported only 4 stems with more than 2 mm of subsidence at the 1-year follow-up. In a prospective study, 122 similar stems were followed for 5 years and did not show subsidence greater than 2 mm (Klein et al. 2009), so the lack of initial stability probably cannot be explained by the coating material.

According to Karrholm et al. (1994), our RSA results are predictive of a less good long-term outcome with the risk of a high rate of stem revisions. During the observation period in our study, 2 of 50 patients underwent revision surgery due to implant loosening.

A learning curve can be expected when new implants are introduced. Before the start of the investigation, both surgeons completed 5 THAs using the VerSys stem to gain knowledge of this specific implant. It would be reasonable to assume that undersized stems led to distal migration. However, as mentioned, all preoperative radiographs were properly templated and all postoperative radiographs were carefully examined by the authors (who did not perform the operations) and found to be within the normal standard of THA with regard to prosthesis size, valgus/varus position, and fit and fill of the implant at the diaphyseal and metaphyseal regions of the femur. Furthermore, the surgical technique was performed according to the manufacturer's instructions by 2 experienced orthopedic surgeons with no difference in outcome parameters between surgeons. Also, no pattern was seen regarding migration value and time after the start of the investigation, which does not support the idea of a prolonged learning curve. This indicates that factors other than the operation technique may have caused the high rate of distal migration.

We acknowledge that our study had several limitations. First, the number of patients at the 2-year follow-up was below the cutoff limit for the calculated sample size, for reasons already mentioned. The validity of the comparison of the 2 stem types can therefore be questioned, owing to the possibility of a type-II error. Secondly, the tantalum markers were mounted by drilling a hole in the implant and fixed with a 0.2-mm larger tap using a press-fit technique. So the markers were not welded together with the implant and the stem had therefore not been exposed to severe heating. However, this procedure requires that the sealing of the sterile packing has to be broken and the implant has to be resterilized, which could theoretically alter the physical properties of the stem.

The alteration of the stems to make RSA measurements possible may also affect the migration—especially migration in the distal direction. This may happen particularly in cases where the markers attached to the medial side have made contact with the calcar bone, and the distal migration may be underestimated. In an attempt to avoid this problem, new marker-free RSA systems are being developed and currently tested (Baad-Hansen et al. 2007).

Ideally, new implant designs should be tested regarding migration rate in a small number of patients over a 2-year observation period before implementation in the daily clinic. The hypothesis in the study was that stems with extended coating would achieve a higher degree of fixation over a larger area, leading to better initial stability. This could not be demonstrated, and surprisingly we found a massive amount of migration of both stems that could lead to premature revision. We believe that additional follow-up is essential. We also believe that this study is a good example of why RSA should be implemented as a standard test when new implants are introduced.
